# The Usefulness of Microencapsulated Sodium Butyrate Add-On Therapy in Maintaining Remission in Patients with Ulcerative Colitis: A Prospective Observational Study

**DOI:** 10.3390/jcm9123941

**Published:** 2020-12-04

**Authors:** Marta Vernero, Federico De Blasio, Davide Giuseppe Ribaldone, Elisabetta Bugianesi, Rinaldo Pellicano, Giorgio Maria Saracco, Marco Astegiano, Gian Paolo Caviglia

**Affiliations:** 1 Department of Internal Medicine, San Matteo Hospital, 27100 Pavia, Italy; marta.vernero@gmail.com; 2 Department of Medical Sciences, University of Turin, 10124 Turin, Italy; deblasio.fe@gmail.com (F.D.B.); elisabetta.bugianesi@unito.it (E.B.); giorgiomaria.saracco@unito.it (G.M.S.); gianpaolo.caviglia@unito.it (G.P.C.); 3 Unit of Gastroenterology, Città della Salute e della Scienza di Torino-Molinette Hospital, 10126 Turin, Italy; rinaldo_pellican@hotmail.com (R.P.); marcoastegiano58@gmail.com (M.A.)

**Keywords:** calprotectin, complementary therapy, IBD, maintaining therapy, SCFA

## Abstract

Butyrate is a short-chain fatty acid that plays a key role in maintaining gut homeostasis as well as the integrity of the intestinal barrier. In the present study, we investigated the effect of oral microencapsulated sodium butyrate (BLM) administration in maintaining remission and improving residual symptoms and inflammatory markers in a population of patients with ulcerative colitis (UC). Forty-two patients with UC in clinical remission were enrolled in the study. Three patients were lost to follow up; 39 patients (18 treated with BLM add-on therapy and 21 with standard mesalamine only) that reached 12 months of follow up were included in the final analysis. Therapeutic success (defined as Mayo partial score ≤ 2 and faecal calprotectin (FC) < 250 µg/g at 12 months of follow up) was achieved in 25 patients (64.1%); 15/18 (83.3%) in BLM group and 10/21 (47.6%) in control group (*p* = 0.022). Consistently, 13/18 patients (72.2%) receiving BLM improved residual symptoms compared to 5/21 patients (23.8%) in control group (*p* = 0.003). FC values significantly diminished from the baseline to the end of follow up in patients that received BLM, while FC values remained almost stable in the control group. In conclusion, oral BLM supplementation appears to be a valid add-on therapy in order to maintain remission in patients with UC. Further randomized, placebo-controlled, double-blind clinical trials are needed to validate our results on a larger population or cohort of patients.

## 1. Introduction

Ulcerative Colitis (UC) and Crohn’s Disease (CD) are inflammatory bowel diseases (IBD) characterized by a chronic-remittent clinical course [1]. While CD could affect any part of the gastrointestinal tract, UC usually involves the rectum and sigmoid colon, possibly extending up to the caecum [2]. Although the aetiopathogenesis of IBD is not completely known, several genetic, immunological, environmental factors, and intestinal permeability are implied in the onset of the disease [3,4,5,6]. In addition, microbiota composition and its interaction with the mucosal immune system are thought to contribute to the pathogenesis of IBD [7,8].

Short-chain fatty acids (SCFAs), such as acetate, propionate, and butyrate, are the main end-products of microbiome fermentation; in particular, butyrate plays a key role in maintaining gut homeostasis as well as intestinal barrier integrity [9,10]. Studies in vivo and in vitro have shown that butyrate is able to exert anti-inflammatory properties through the inhibition of nuclear factor κ-B (NF-κB) [11,12,13], reducing the expression of proinflammatory genes, including those encoding for proinflammatory cytokines, chemokines, and adhesion molecules [14].

A recent study involving patients with IBD pointed out- an association between butyrate levels and response to biologic therapy [15]; further, several pieces of evidence suggested a beneficial effect of probiotics/prebiotics supplementation as add-on therapy in UC patients [16,17]. Facchin and colleagues conducted a double-blind, placebo-controlled pilot study investigating the effect of oral microencapsulated-sodium-butyrate (BLM) administration on microbiome composition among a population of 49 IBD patients (19 CD and 30 UC). IBD patient’s microbiome was compared to that of 18 healthy volunteers. It was found that exogenous butyrate increased butyrate-producing bacteria, which in turn increased endogenous butyrate production, contributing to gut homeostasis [18]. Finally, in patients with active UC, administration of butyrate in association to standard therapy demonstrated a significant improvement of inflammatory parameters [19,20,21,22,23].

The aim of the present study was to investigate the effect of oral microencapsulated sodium butyrate (BLM) administration in maintaining remission and improving residual symptoms and inflammatory markers in a population of patients with UC.

## 2. Materials and Methods

### 2.1. Study Design and Patients

This was an observational, single-center, prospective cohort study conducted at the outpatient clinic of the Unit of Gastroenterology of “Città della Salute e della Scienza di Torino—Molinette” Hospital, Turin, Italy, between January 2019 and February 2020.

Patients with at least 18 years of age, histologically confirmed diagnosis of UC according to ECCO criteria [2], and in clinical remission were eligible for this study. For the case group, an additional inclusion criterion was being prescribed BLM (Butyrose^®^, Sila s.r.l., Noale, VE, Italy) supplementation immediately prior to the inclusion in the study. Since mesalazine is the standard treatment to maintain remission in UC, the treating physician (M.A.) decided to add BLM or not in patients with UC in clinical remission according to patients’ preference (willingness to take new pills and economic availability). The exclusion criteria were clinically active disease (Mayo Partial Score (MPS) ≥ 3) [24], disease extension limited to the rectum, previous colectomy or surgical resections, treatment with systemic or topic steroids, and antibiotics or pre/probiotics therapy in the 30 days before enrolment, and antibiotics or pre/probiotics therapy during the whole follow-up period. UC limited to the rectum was excluded because topical therapy is the cornerstone of its therapy and because systemic inflammation biomarkers are typically not altered. All patients received standard therapy with oral mesalamine 2400 mg/day during the 12 months follow-up. Patients on BLM therapy received a dose of two capsules/day for 12 months (500 mg of BLM for each capsule), while the control group was followed for 12 months with no therapy modifications. All the patients were clinically assessed at baseline (T0), at six months (T1), and one year (T2) after enrolment. At each time-point, we collected clinical data (disease activity by MPS, quality of life and abdominal pain) and biochemical parameters, including C-reactive protein (CRP), erythrocyte sedimentation rate (ESR) (useful in UC not limited to the rectum, since endoscopic data were not available in this observational study), and fecal calprotectin (FC). Disease-related quality of life was evaluated through the self-administration of the Short Inflammatory Bowel Disease Questionnaire (SIBDQ) [25]; the minimum score for SIBDQ is 10 points, indicating very poor quality of life, while the maximum score is 70 points, indicating an optimal quality of life. Abdominal pain was evaluated using a visual analogue scale (VAS) [26]; a VAS score = 0 corresponded to no pain, while a VAS score = 10 indicated extreme pain.

Study procedures were compliant with the principles of the Declaration of Helsinki. All patients gave their written informed consent, and the study was approved by the Ethics Committee of the Città della Salute e della Scienza—University Hospital of Turin (approval code 0056924).

### 2.2. Outcomes

The primary outcome of the study was maintaining disease remission after 12 months of oral BLM supplementation. Treatment was defined as successful for MPS ≤ 2 and FC < 250 µg/g at T2, without the necessity to adjust therapy during the follow-up.

The secondary outcomes were the improvement of subjective symptoms (quality of life and abdominal pain) and inflammatory parameters (CRP, ESR, and FC) at 6 and 12 months from enrolment. Patients showing any decrease in VAS score and an increase in SIBDQ score at T1 and T2 were considered clinically improved. Patients whose VAS was increased or SIBDQ was decreased were not considered clinically improved. Patients showing concomitant reduction of CRP, ERS, and FC levels at T1 and T2 were considered biochemically improved; those who did not were considered biochemically non-improved.

Patients lost to follow-up were not considered in the final analysis (per-protocol analysis).

### 2.3. Statistical Analysis

Continuous variables were reported as median and range or 95% confidence interval (CI) or mean ± standard deviation (SD) according to data distribution. Normality was checked using the D’Agostino–Pearson test. Categorical variables were reported as number and percentage. Comparison of continuous variables between independent groups was performed using either a Mann–Whitney test or a Students’ *t*-test; comparison between paired measurements was performed by using either a Wilcoxon test or a Students’ *t*-test. Evaluation of the kinetics of continuous variables was performed by a Friedman test or a repeated-measures analysis of variance (ANOVA), where appropriate. Regarding the dichotomous categorical variable, a chi-squared (χ^2^) test or a McNemar test were performed for unpaired or paired analysis, respectively.

All statistical analyses were performed by using MedCalc^®^ v.18.9.1 (MedCalc Software Ltd., Ostend, Belgium), and a *p*-value ≤ 0.05 was considered statistically significant.

## 3. Results

A total of 42 patients with UC in remission met the inclusion criteria and were enrolled in the study; 21 out of 42 (50%) received BLM add-on therapy. The principal baseline characteristics of the study population are reported in Table 1.

At baseline, no differences were observed between patients treated with or without BLM according to age, gender, disease activity, quality of life, and abdominal pain. Regarding inflammatory biomarkers, baseline ESR and FC values were similar between the two groups of patients (*p* = 0.290 and *p* = 0.200, respectively), while CRP values were slightly lower in patients treated with BLM (*p* = 0.034) but below the upper limit of normal in both groups.

**Figure 1:**
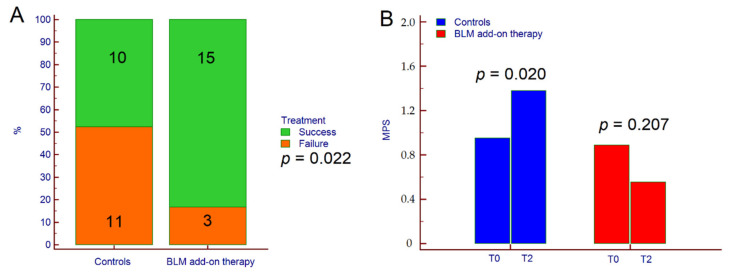
Therapeutic success (**A**) and variation of MPS from T0 to T2 (**B**) in patients treated with BLM add-on therapy and in the control group. The *p*-value in (**A**) was calculated by χ^2^ test while the *p*-values in (**B**) were calculated by paired Students’ *t*-test. Abbreviations: chi-squared (χ^2^), microencapsulated sodium butyrate (BLM), Mayo partial score (MPS), baseline (T0), and end of follow-up (T2).

As per the protocol, three patients receiving BLM were lost to follow-up; therefore, they were not included in the final analysis. Concerning primary outcome, therapeutic success was achieved in 25 patients (64.1%); 15 out of 18 (83.3%) in the BLM group, and 10 out of 21 (47.6%) in the control group (*p* = 0.022) (Figure 1A). Consistently, MPS significantly increased from 0.8 ± 0.7 at T0 to 1.4 ± 1.3 at T2 in the control group (*p* = 0.020) but not in patients receiving BLM (1.0 ± 0.7 at T0 vs. 0.6 ± 0.6 at T2, *p* = 0.207) (Figure 1B).

**Figure 2:**
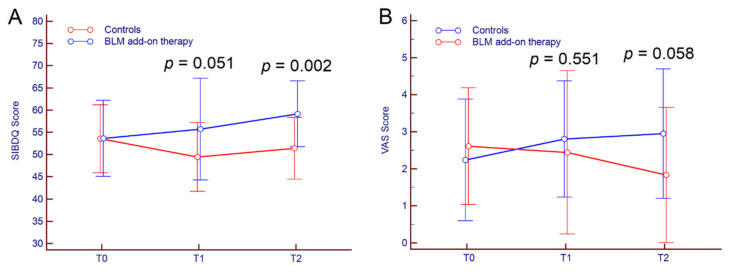
Kinetics of SIBDQ (**A**) and VAS (**B**) scores from T0 to T2 in UC patients treated with BLM add-on therapy and in the control group. Comparison between groups at different time-points was performed by Students’ *t*-test. Abbreviations: microencapsulated sodium butyrate (BLM), Short Inflammatory Bowel Disease Questionnaire (SIBDQ), visual analogue scale (VAS).

Regarding secondary outcomes, subjective improvement (SIBDQ + VAS) after six months of follow-up was achieved in 11 out of 18 patients (61.1%) receiving BLM vs. three out of 21 (14.3%) in the control group (*p* = 0.003); at 12 months of follow up, 13 out of 18 patients (72.2%) receiving BLM improved SIBDQ and VAS scores compared to five out 21 patients (23.8%) in the control group (*p* = 0.003). Indeed, in patients treated with BLM add-on therapy, the mean SIBDQ score stepwise increased from T0 to T2 (*p* = 0.023) while it decreased significantly in the control group (*p* = 0.018). Similarly, VAS score raised significantly from T0 to T2 in the control group (*p* = 0.019), while distinctly increased in BLM patients, even though the result was not statistically significant (*p* = 0.154). SIBDQ and VAS scores from T0 to T2 are reported in Table 2 and depicted in Figure 2.

Concomitant improvement of all the inflammatory markers was observed at T1 in 11 out of 18 patients (61.1%) of the BLM treatment group and in three out of 21 patients (14.3%) of the control group at T1 (*p* = 0.003). After 12 months of add-on therapy, 13 out of 18 patients (72.2%) who completed follow-up achieved a biochemical improvement compared to five out of 21 patients (23.8%) in the control group (*p* = 0.003).

**Figure 3:**
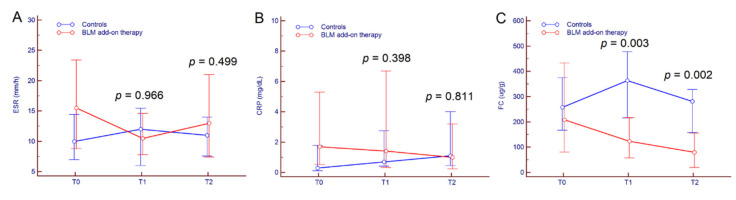
Kinetics of ESR (**A**), CRP (**B**), and FC (**C**) from T0 to T2 in UC patients treated with BLM add-on therapy and in the control group. Comparison between groups at different time-points was performed by a Mann–Whitney test. Abbreviations: microencapsulated sodium butyrate (BLM), fecal calprotectin (FC), C reactive protein (CRP), and erythrocyte sedimentation rate (ESR).

Among inflammatory markers, no significant variation was observed for ESR and CRP, neither within groups nor between groups (Table 2, Figure 3A,B). Conversely, FC values distinctly diminished from T0 to T2 in patients that received BLM while they remained almost stable in the control group; as a result, median FC values were significantly different between the two groups of patients both at T1 (*p* = 0.003) and at T2 (*p* = 0.002) (Figure 3C).

## 4. Discussion

The main finding of our study was that the majority of patients (83.3%) receiving BLM add-on therapy maintained clinical remission compared to the 47.6% of patients who underwent standard therapy with mesalamine only. Furthermore, the rates of clinical and biochemical improvement were significantly higher in the former compared to the latter. As a matter of fact, BLM has a strong anti-inflammatory and antioxidant activity, as well as a protective effect on the gut barrier; indeed, butyrate is an energy source for intestinal epithelial cells and contributes to their integrity [27]. As recently suggested, BLM was able to influence gut microbiome composition reducing inflammation of the colon [18].

UC is characterized by alternating periods of recurrence and remission; with medication, the yearly relapse rates have been estimated between 12% and 58% [28,29,30]. It has been shown that probiotic treatment for one year led to lower recurrence rates in patients with UC aged 12 to 16 years compared to a control group (21% vs. 73%) [31]. Another study showed that supplementation with *Lactobacillus* species in addition to standard treatment reduced the risk of relapse in patients with UC [32]. Furthermore, the combined administration of mesalamine and *Lactobacillus reuteri* ATCC 5573 reduced mucosal inflammation in children with UC localized to the rectum [33]. Here, we observed that after 12 months of BLM administration, most of the patients maintained remission both in terms of disease activity (MPS ≤ 2) and in terms of negative FC levels (<250 µg/g) [34,35], while in the control group less than one-half of the patients maintained remission. International guidelines indicate that the only alternative to mesalamine in maintaining UC remission is *Escherichia coli* Nissle 1917 (EcN) [2]; however, the results of our study support the possible use of BLM as add-on therapy in these patients.

Concerning secondary outcomes, the quality of life in patients that underwent 12 months of BLM therapy significantly improved, and the difference with patients treated only with mesalamine became progressively more prominent from T1 to T2. Concurrently, we did not observe any improvement of VAS score in BLM-treated patients, while among the control group, abdominal pain worsened during the follow-up period. These findings are interesting since, up to now, there has been no clear evidence concerning the optimal duration of BLM supplementation in UC patients in remission; our study suggests that BLM should be prescribed for a long period in order to achieve the best effect.

After 12 months of add-on therapy, 72.2% of patients who completed follow-up achieved a biochemical improvement compared to the 23.8% in the control group, suggesting that long-term BLM add-on therapy may modulate gut inflammation, possibly through microbiome modification [8,17]. Remarkably, FC values significantly diminished in patients treated with BLM compared to those treated with standard mesalamine maintaining therapy. This finding is particularly relevant because FC is widely used to predict the clinical course in patients with UC, given its strong association with disease relapse. Indeed, patients with clinically inactive disease and high FC levels have an 80% chance of clinical relapse in the next six months, and two consecutively elevated FC values are highly associated with endoscopic relapse [34].

The main limitation of this study is the observational design; therefore, the lack of randomization prevented us from control for possible bias related to patient characteristics that may have an impact on the outcome. Furthermore, the limited sample size may represent an additional limitation of the present study. Nevertheless, baseline patients’ characteristics were broadly comparable, and all patients were treated by the same IBD expert clinician (M.A.) that prescribed BLM add-on therapy following the same criteria for all patients. Therefore, we believe that the results of our study are promising and set the basis for controlled clinical trials with broader case studies.

## 5. Conclusions

In conclusion, oral BLM supplementation appeared a valid add-on therapy in order to maintain remission in patients with UC. Patients treated with BLM in combination with standard mesalamine achieved higher clinical improvement rates and experienced an amelioration of inflammatory markers, especially FC. Further randomized, placebo-controlled, double-blind clinical trials are needed to validate our results on a larger population of patients with UC in remission.

## Figures and Tables

**Table 1:** Baseline characteristics of the overall study population and according to the BLM add-on therapy.

Characteristics	Total Population	BLM Add-On Therapy Group	Control Group	*p*-Value
Patients, *n* (%)	42	21	21	
Age (years), median (range)	50 (25–75)	53 (26–75)	47 (25–75)	0.753
Gender (M/F)	30/10	17/4	13/8	0.177
MPS, mean ± SD	0.9 ± 0.8	1.0 ± 0.7	0.8 ± 0.7	0.571
Bristol stool scale, mean ± SD	4.4 ± 0.9	4.2 ± 1.0	4.6 ± 0.8	0.181
SIBDQ score, mean ± SD	53 ± 8	54 ± 8	53 ± 8	0.861
VAS score, mean ± SD	2.3 ± 1.6	2.2 ± 1.6	2.3 ± 1.7	0.926
ESR (mm/h), median (95% CI)	11 (8–15)	10 (7–14)	13 (8–19)	0.290
CRP (mg/dL), median (95% CI)	0.7 (0.3–2.0)	0.3 (0.1–1.8)	1.6 (0.5–5.2)	0.034
FC (µg/g), median (95% CI)	226 (167–309)	258 (167–375)	207 (86–321)	0.200

Abbreviations: microencapsulated sodium butyrate (BLM), confidence interval (CI), male (M), female (F), fecal calprotectin (FC), C reactive protein (CRP), erythrocyte sedimentation rate (ESR), Mayo partial score (MPS), standard deviation (SD), Short Inflammatory Bowel Disease Questionnaire (SIBDQ), visual analogue scale (VAS).

**Table 2:** Quality of life, abdominal pain, and inflammatory markers from T0 to T2 in the overall population of patients with UC and according to the BLM add-on therapy.

Parameters	*n*	T0	T1	T2	*p*-Value *
SIBDQ score, mean ± SD	39	54 ± 8	52 ± 10	55 ± 8	0.104
BLM group	18	54 ± 9	56 ± 11	59 ± 7	0.023
Control group	21	54 ± 8	49 ± 8	51 ± 7	0.018
VAS score, mean ± SD	39	2.4 ± 1.6	2.6 ± 1.9	2.4 ± 1.8	0.593
BLM group	18	2.6 ± 1.6	2.4 ± 2.2	1.8 ± 1.8	0.154
Control group	21	2.2 ± 1.6	2.8 ± 1.6	3.0 ± 1.7	0.019
ESR (mm/h), median (95% CI)	39	12 (9–15)	11 (8–14)	11 (9–14)	0.612
BLM group	18	16 (9–23)	11 (8–15)	13 (7–21)	0.019
Control group	21	10 (7–14)	12 (6–15)	11 (8–14)	0.464
CRP (mg/dL), median (95% CI)	39	0.8 (0.3–2.0)	0.8 (0.5–3.7)	1.0 (0.5–3.2)	0.410
BLM group	18	1.7 (0.5–5.3)	1.4 (0.3–6.7)	1.0 (0.2–3.2)	0.274
Control group	21	0.3 (0.1–1.8)	0.7 (0.4–2.8)	1.1 (0.5–4.0)	0.002
FC (µg/g), median (95% CI)	39	233 (167–319)	220 (132–366)	161 (103–256)	0.195
BLM group	18	209 (81–434)	124 (57–217)	80 (57–217)	0.085
Control group	21	258 (167–375)	364 (216–478)	281 (157–328)	0.051

* *p*-values were calculated by repeated-measures analysis of variance (ANOVA) for SIBDQ and VAS score, and by Friedman test for ESR, CRP and FC. Abbreviations: confidence interval (CI), male (M), female (F), fecal calprotectin (FC), confidence interval (CI), C reactive protein (CRP), erythrocyte sedimentation rate (ESR), standard deviation (SD), Short Inflammatory Bowel Disease Questionnaire (SIBDQ), and visual analogue scale (VAS).
